# Pentynyl Ether of β-Cyclodextrin Polymer and Silica Micro-Particles: A New Hybrid Material for Adsorption of Phenanthrene from Water

**DOI:** 10.3390/polym9010010

**Published:** 2017-01-04

**Authors:** Jae Min Choi, Daham Jeong, Eunae Cho, Jae-Hyuk Yu, Muhammad Nazir Tahir, Seunho Jung

**Affiliations:** 1 Center for Biotechnology Research in UBITA (CBRU), Institute for Ubiquitous Information Technology and Applications (UBITA), Konkuk University, 120 Neungdong-ro, Gwangjin-gu, Seoul 05029, Korea; jm.choi.25@gmail.com (J.M.C.); 82goodgirl@hanmail.net (E.C.); 2 Department of Systems Biotechnology, Microbial Carbohydrate Resource Bank (MCRB) & Center for Biotechnology Research in UBITA (CBRU), Konkuk University, 120 Neungdong-ro, Gwangjin-gu, Seoul 05029, Korea; amir@konkuk.ac.kr; 3 Department of Bacteriology, The University of Wisconsin-Madison, Madison, WI 53706, USA; jyu1@wisc.edu; 4 Nanoqam, Department of Chemistry, University of Quebec at Montreal, P.O. Box 8888, Succ. Centre-ville, Montreal, QC H3C 3P8, Canada

**Keywords:** pentynyl ethers of beta-cyclodextrin, silica micro-particles, hybridization, polycyclic aromatic hydrocarbons, inclusion complex

## Abstract

A new hybrid material for the removal of polycyclic aromatic hydrocarbons (PAH) from water was prepared by the polymerization of pentynyl beta-cyclodextrin (PyβCD) and silica micro-particles (SMP). Phenanthrene, being one of the important members of the PAH family and a potential risk for environmental pollution, was selected for this study. Results show that phenanthrene removal efficiency of the SMP was improved significantly after hybridization with PyβCD-polymer. Approximately 50% of the phenanthrene was removed in the first 60 min and more than 95% was removed in less than 7 h when 25 mL of the 2 ppm aqueous phenanthrene solution was incubated with the 100 mg of SMP-PyβCD-polymer material. Infrared spectroscopy and thermal gravimetric analysis show that the enhanced efficiency of the SMP-PyβCD-polymer compared to the unmodified SMP was due to the formation of the inclusion complexation of phenanthrene with the PyβCD. These results indicate that SMP-PyβCD polymers have a potential to be applied as molecular filters in water purification systems and also for waste water treatment.

## 1. Introduction

Polycyclic aromatic hydrocarbons (PAH) are considered one of the most toxic and widespread pollutants which cause severe health problems not only for human beings, but also for other living organisms [1,2]. Coke-oven gas plants, refineries, and related chemical industries are the main sources of PAH emission into the environment [3]. Most of the PAH-producing industries are located near urban areas and, thus, represent a considerable public health threat [3]. Due to their persistent nature and toxic, mutagenic, and carcinogenic effects, the remediation of PAH-contaminated water is an important environmental issue. However, the low-water solubility of these components makes their removal from the contaminated water very difficult [1,2]. Phenanthrene, being one of the most important PAHs, composed of three fused benzene rings, was selected for investigation in this study.

In previous years, a number of different adsorbents, like nanofibers, silica gel, porous nanoparticles, and other similar materials have been developed for the removal of PAHs [4,5,6] but most of such materials could not be applied at large scale for water purification due to their limited efficiency, high production cost or due to the involvement of complex synthetic procedures. Very low solubility of the PAH in water is another reason for the limited use of these kind of materials. However, there are a number of reports indicating that beta-cyclodextrin (βCD) could increase the solubility of aromatic compounds [7,8] and removal efficiency enhanced several times [3].

βCD is a cyclic heptasaccharide composed of seven glucose units joined ‘‘head to tail’’ by an α-(1–4) glucopyranose unit link. βCD has a toroid-shaped molecular structure with a relatively hydrophobic interior cavity at the center of its molecular arrangement, which can make a non-covalent reversible host–guest complexation with many molecules having a phenyl ring [9,10,11,12]. Due to host-guest inclusion complexation, βCD is applied in pharmaceuticals, foods, cosmetics, textile, wound odor absorbants, composites, gene delivery, and in the other related industries [10,12,13,14,15,16,17,18,19,20,21,22]. Additionally, water filtration and purification systems are another field of application for βCD molecules owing to their potential of encapsulating hazardous organic molecules [23,24,25,26]. Moreover, βCD is preferred over the other adsorbents because it is biocompatible, environmentally friendly, non-toxic, non-hygroscopic, and chemically stable [27].

Depending upon the target applications, βCD could be used in various forms, like chemical derivatives and polymers [10,12,25]. Polymerization of βCD leads to the formation of a 3D network containing both hydrophobic and hydrophilic cavities [28]. Soluble hyper-cross-linked βCD polymers are of great interest, e.g., in the encapsulation of organic molecules and inorganic ions [29], but could not be applied in removing pollutants from water. Water solubility of βCD or its derivatives would restrict its applications for water purification systems due to the possibility of leaching from the surface during the filtration process [30]. Water insoluble βCD polymers have their own advantages in specific applications e.g., nanosponges and hydrogels, for controlled drug delivery systems [15], encapsulation, and controlled release of agricultural chemicals [31], and for environmental applications [25]. Thus, natural CDs are often chemically modified to synthesize novel insoluble CD-derivatives. The patents published by Martel et al. [32] and Trotta et al. [33] can be consulted for the use of carboxylic acids and pyromellitic dianhydride, respectively, as agents to cross-linking CDs. Other cross-linking agents like epichlorohydrin (ECH), ethylene glycol diglycidyl ether, glutaraldehyde, benzoquinone, isocyanates, and carbodiimidazole, etc., have also been reported [25,28,34]. In this list, ECH, due to its low cost, high reactivity and long bridges by self-polymerization, is the most commonly used cross-linking agent to prepare CD polymers [35,36]. However, low cross-linking density of ECH-based βCD-polymers tends to increase their water-solubility due to the long bridged chains of ECH that leads to disruptions in the intermolecular hydrogen bonding between βCD and water molecules [37]. Therefore, we suggest applying a hybrid material prepared by the high-density polymerization of βCD in the presence of silica micro-particles (SMP). SMP could, itself, be used for the adsorption of PAHs [38] but its efficiency could be enhanced significantly by its hybridization with βCD. Instead of using a simple βCD polymer, we introduced pentynyl groups onto the βCD surface (→PyβCD, **2**) by its etherification with pentynyl chloride followed by the polymerization and hybridization with SMP. Native βCD is hydrophilic in nature due to the presence of a large number of –OH groups while its pentynylation (PyβCD, **2**) leads to the introduction of carbon rich –CH2–CH2–CH2–C≡CH residues, which introduce hydrophobic properties. The hydrophobic character of the PyβCD (**2**) could be varied by varying the degree of substitution (DS = average number of pentynyl groups introduced per glucosyl unit, max = 3). In addition, terminal acetylenic groups act as hydrogen bonding donors and acceptor, as well [39]. Thus, PyβCD (**2**) enables several types of weak cooperative interactions [39] that could possibly support efficient PAH adsorption. Moreover, βCD polymer is normally soluble in water and, thus, not suitable for water purification systems to remove PAHs, while PyβCD-polymer is insoluble due to the hydrophobic character introduced by the pentynyl groups. Herein, we report a simple process for the preparation of pentynyl βCD (PyβCD, **2**) and its polymerization (Poly-PyβCD, **3**) using epichlorohydrin in the presence of SMP to prepare a hybrid material (Poly-SMP-PyβCD, **4**) as a potential candidate to adsorb phenanthrene from the water.

## 2. Experimental

### 2.1. Materials

Silica micro-particles (av. 4 µm in diameter) were purchased from Polyscience Inc., Warminster, PA, USA. βCD (≥97%), acetone (99.9%), dimethyl sulfoxide (DMSO, 99.9%), *N*,*N*-dimethyl formamide (99.8%), pentynyl chloride (PyCl, 98%), epichlorohydrin (≥99%), acetonitrile (High Perfomance Liquid Chromatography (HPLC) plus, ≥99.9%), methanol (≥99.9%), hexane (≥95%), and methyl lithium (1.6 M in diethyl ether) were purchased from Sigma–Aldrich, St. Louis, MO, USA. PyβCD was obtained from Microbial Carbohydrate Resource Bank at Konkuk University, Seoul, Korea. Water used in all the experiments was purified using Direct-Q Millipore water purification system from Sam Woo Co., Ltd., Gyeonggi, Korea.

### 2.2. Pentynylation of βCD (PyβCD, **2**)

**Scheme 1:**
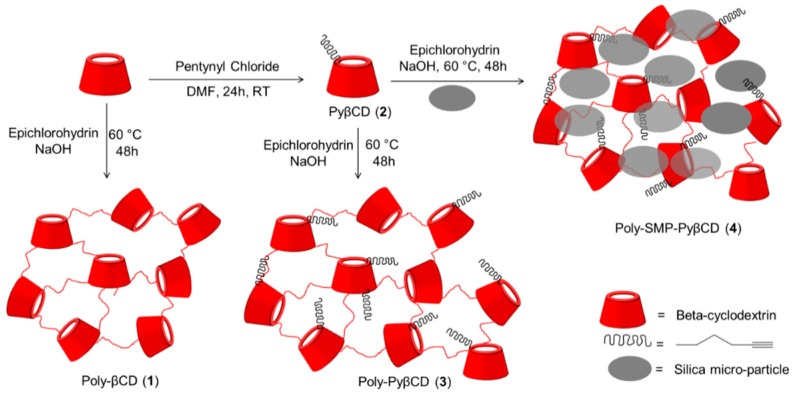
Schematic representation for the synthesis of βCD polymer (Poly-βCD, **1**), pentynyl ether of βCD (PyβCD, **2**), polymer of PyβCD (Poly-PyβCD, **3**), and hybridization with SMP (Poly-SMP-PyβCD, **4**).

βCD (5 g, 4.40 mmol) was dissolved in *N*,*N*-Dimethylformamide (100 mL) followed by the addition of Li-dimsyl (3.88 g, 46.25 mmol, 0.5 eq./OH) prepared according to the procedure as described earlier [27] and stirred for 1 h. PyCl (4.74 g, 46.25 mmol, 0.5 eq./OH) was also added and the mixture was stirred at room temperature for 24 h (Scheme 1). Solvent was evaporated under reduced pressure and residue was suspended in water and precipitated in acetone. The product was recrystallized from cold water and dried under vacuum as a white powder (7.12 g). ^1^H-NMR (500 MHz, DMSO-*d*_6_) δ = 1.68 (m, 2H), 2.24 (m, 2H), 2.71 (s, 1H), 3.31–3.61 (m, 40H), 4.13 (m, 1H), 4.27–4.29 (m, 1H), 4.33 (t, 1H), 4.41 (m, 2H), 4.48 (t, 3H), 4.72 (d, 2H), 4.80 (m, 5H), 5.02 (m, 2H), 5.64–5.73 (m, 14H).

### 2.3. Polymerization of PyβCD (**2**→Poly-PyβCD, **3**) in the Presence of Silica Micro-Particles

In a typical procedure, 2 g of PyβCD (**2**) was added into 40 mL of water followed by the addition of 6.8 mL of the 20% NaOH. The mixture was stirred at 60 °C until a clear solution was obtained. Two grams of SMP was added and stirring of the mixture continued for 1 h. After that, 162.84 mg (1.76 mmol) of epichlorohydrin (ECH) was also added into the reaction mixture and continued stirring at 60 °C. After 48 h, reaction was stopped and product was purified by centrifugation (4000 rpm × 20 min × 4 times), washed with methanol, hexane, and acetone (three times with each solvent) and dried in open air to obtain the hybrid composite material (Poly-SMP-PyβCD, **4**, 3.94 g). For comparison, βCD and PyβCD (**2**) was also polymerized without SMP following the same procedure as described above and precipitated in acetone (Scheme 1).

### 2.4. Phenanthrene Removing Studies

Phenanthrene was dissolved in minimum amount of the acetonitrile and diluted in water and analyzed by HPLC. The calibration curve of phenanthrene was prepared in preliminary studies by analyzing phenanthrene solutions from 1–6 ppm prepared by the serial dilution of the same stock solution. The curve showed linearity with *R*^2^ ≥ 0.99. In the general procedure of phenanthrene removal from the aqueous solution, 100 mg of the Poly-SMP-PyβCD (**4**) was incubated with the 25 mL of the 2 ppm phenanthrene solution and samples (250 µL each time) were drawn after every 20 min and analyzed by HPLC. The results (in terms of peak areas) were adapted to the calibration curve prepared in the preliminary studies.

### 2.5. Characterization of the Materials

Scanning electron microscopy (SEM) images were recorded with a JEOL JSM-6380 scanning electron microscope (JEOL Ltd., Tokyo, Japan). NMR spectra were recorded with a Bruker Avance III HD 300 MHz spectrometer (Bruker, Billerica, MA, USA). Proton chemicals shifts are reported in ppm relative to the solvent signals. Thermal gravimetric analysis (TGA) was performed by means of a TG\TDA6200 thermal analyzer (Seiko instrument Inc., Chiba-shi, Japan), under a 100 mL·min^−1^ nitrogen stream and 5 °C/min heating rate. X-ray photoelectron spectra (XPS) were recorded using a XPS PHI 5600-ci (Physical Electronics, Eden Prairie, MN, USA). Al-standard (1486.6 eV) was employed as the anode for a wide-scan spectra at 300 W). High-resolution spectra were obtained with an Mg anode (1253.6 eV) at 150 W. The analyses were performed without charge compensation (neutralizer) at an angle of 45° with respect to the surface. Infrared (IR) spectra were recorded using an ATR-IR spectrometer (Model 550, Magna Nicolet, Madison, WI, USA). HPLC studies were conducted on the Agilent 1200 Series HLPC system (Agilent Technologies, Santa Clara, CA, USA) equipped with a Zorbax Eclipse XDB-C_18_ column (150 mm × 4.6 mm, 5 µm particle size) and acetonitrile as the mobile phase at a flow rate of 0.4 mL/min. Each time a 10 µL sample was injected and phenanthrene was detected at 254 nm wavelengths.

## 3. Results and Discussion

### 3.1. Synthesis and Characterization of the Materials

Poly-βCD (**1**), PyβCD (**2**), Poly-PyβCD (**3**), Poly-SMP-PyβCD (**4**) were prepared as shown in Scheme 1 and described in the experimental section. All materials were characterized by SEM, XPS, IR spectroscopy, and TGA.

### 3.2. Scanning Electron Microscopy (SEM)

**Figure 1:**
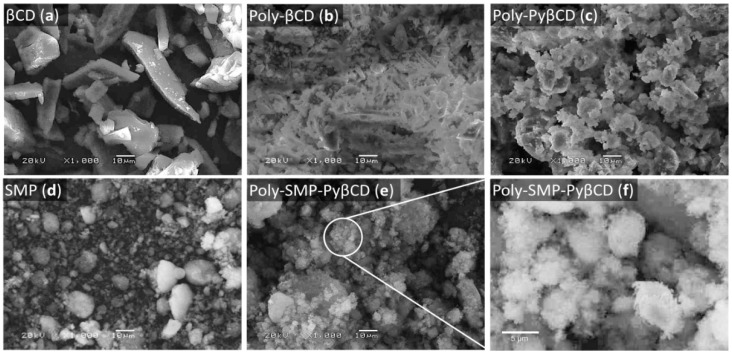
SEM images of the βCD (**a**); Poly-βCD (**b**); Poly-PyβCD (**3**, **c**); silica micro-particles (SMP, **d**), and Poly-SMP-PyβCD (**4**, **e**,**f**).

SEM images of the βCD (Figure 1a), Poly-βCD (**1**, Figure 1b), Poly-PyβCD (**3**, Figure 1c), and that of hybrid materials prepared by polymerization of PyβCD in the presence of SMP (**4**, Figure 1e,f) show that morphological changes in the materials appeared at different stages. βCD in the SEM images appears as a flake-like crystalline (Figure 1a) that has the typical morphology of βCD powder [40]. In case of Poly-βCD, it appeared as a soft fibrous material due to the loss of crystallinity of βCD by ECH-polymerization (Figure 1b) [41]. However, Poly-PyβCD (**3**) showed an entirely different appearance and embossed “flora-like” structures were formed on the outer surface of the βCD polymer (Figure 1c). This was probably due to the hydrophobic character induced by the pentynyl groups grafted on the βCD surface. Comparison of the SMP (Figure 1d), and after its hybridization, (Figure 1e) clearly indicates that SMP were entrapped into the PyβCD network during the polymerization process. This phenomenon is clearer at higher magnification (Figure 1f) where silica particles trapped in the soft, snowy structures of the poly-PyβCD network could be easily seen. These results indicate successful coating of the SMP with PyβCD polymer.

### 3.3. X-ray Photoelectron Spectroscopy (XPS)

**Figure 2:**
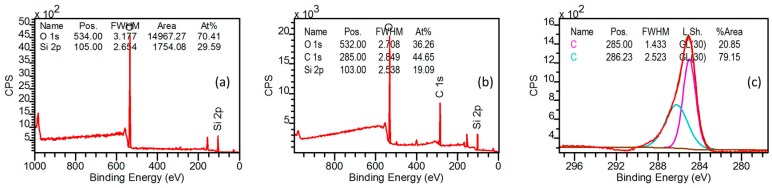
XPS wide scan spectra of unmodified SMP (**a**) and after hybridization with Poly-SMP-PyβCD (**4**, **b**) and narrow-scan of the C1s region after hybridization with Poly-SMP-PyβCD (**4**, **c**).

XPS is important analysis tool for detailed characterization of micro- and nanoparticles [42]. SMP and Poly-PyβCD (**4**) were confirmed by XPS characterization to be successfully hybridized. The wide-scan XPS spectrum (Figure 2a) shows signals only for Si (2p, 105 eV) and O (1s, 534 eV), while that of recorded after hybridization with Poly-SMP-PyβCD (**4**) shows signal for C (2p, 285 eV, Figure 2b) along with Si and O. In elemental composition, a decrease in Si content from 29.59 to 19.09 at% after hybridization also give additional proof that the surface was successfully coated with the Poly-PyβCD (compared to the elemental composition of Si in Figure 2a,b). The narrow-scan XPS spectrum (Figure 2c) of the C1s region displayed a signal at 285.0 eV corresponding to C–C bonds due to the presence of pentynyl groups and a signal at 286.23 eV corresponding to C–O bonds [43] from Poly-PyβCD. The ratio of C–C/C–O is 0.26 and corresponds to 2.78 pentynyl groups per βCD ring and is in agreement with the DS calculated from the NMR spectroscopy.

### 3.4. Applications for the Adsorption of Phenanthrene

**Figure 3:**
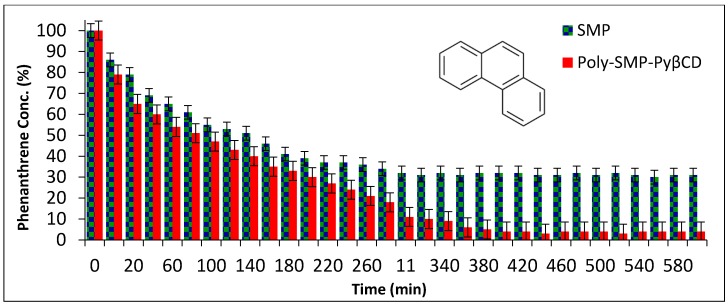
Phenanthrene (structure shown in the graph) adsorption from aqueous solution by the SMP and Poly-SMP-PyβCD (**4**).

To study the phenanthrene adsorption from water, Poly-SMP-PyβCD (**4**) was incubated with the phenanthrene solution with gentle agitation and samples were periodically and analyzed by HPLC. Results represented in Figure 3 show that phenanthrene concentration decreased continuously with the passage of time. For comparison, unmodified SMP was also used in parallel and results (Figure 3) show that, along with Poly-SMP-PyβCD (**4**), unmodified SMP could also adsorb phenanthrene. However, the efficiency of phenanthrene-absorption was significantly improved in the case of the modified SMP (**4**). In the first hour, while SMP adsorbed 35% of the phenanthrene from the solution, this ratio reached 46% for Poly-SMP-PyβCD (**4**). As the time passes, the differences of the adsorbed amount of the phenanthrene between SMP and Poly-SMP-PyβCD (**4**) increases, therefore, the concentration of phenanthrene dropped more rapidly for Poly-SMP-PyβCD (**4**) compared to SMP. After 5 h of incubation, unmodified SMP and Poly-SMP-PyβCD (**4**) removed, respectively, 78% and 89% of the phenanthrene. Afterwards, it remained almost unchanged (Figure 3) for the SMP, while Poly-SMP-PyβCD (**4**) removed about 96% of the phenanthrene in 7 h (Figure 3). The higher phenanthrene removal efficiency of Poly-SMP-PyβCD (**4**) probably originated from the inclusion complexation property of βCD grafted on the SMP surface (**4**). Regarding the inclusion complexation mechanism, it is considered that it mainly occurs due to the hydrophobic interactions between phenanthrene and the βCD cavity. In the presence of repulsive interactions between the hydrophobic phenanthrene and the surrounding aqueous environment, the more favorable interactions between the hydrophobic guest and apolar βCD cavity are the main driving forces for the transfer of phenanthrene molecules from the water to the βCD cavity [13,44]. For comparison, Poly-βCD (**1**), without grafting on to the SMP, was also prepared and used for the removal of phenanthrene from the aqueous solution, but its partial solubility in water makes its recovery very difficult and makes the whole process very complex. On the other hand, PyβCD (**2**), itself, and its polymer (Poly-PyβCD, **3**), are insoluble in water and the hybrid material (**4**) of the Poly-PyβCD (**3**) and SMP is a very efficient and easy to recover from the aqueous solution. Pentynylation of the βCD not only changes its solubility behavior, but also introduces a strong non-polar character making it more applicable without altering its entrapment and removal capacity of phenanthrene molecules. For further confirmation that enhanced phenanthrene removal efficiency of the Poly-SMP-PyβCD (**4**) was due to the encapsulation of phenanthrene into the βCD cavity, the adsorbent was characterized by IR spectroscopy and TGA.

### 3.5. Infrared Spectroscopy

**Figure 4:**
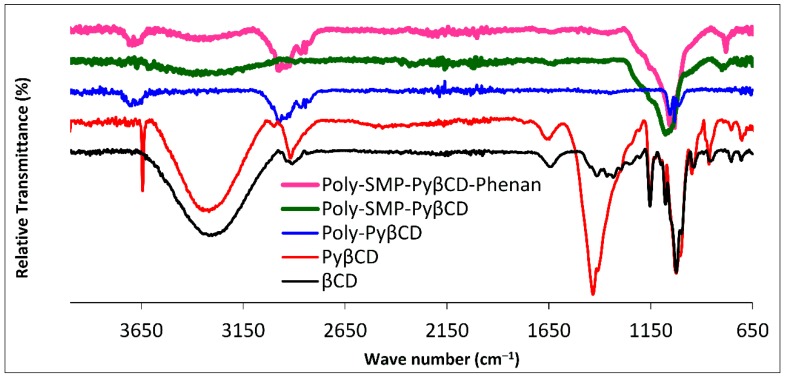
ATR-IR spectra of (respectively from bottom to top) βCD, PyβCD (**2**); Poly-PyβCD (**3**); Poly-SMP-PyβCD (**4**); and that of after making inclusion complex with the phenanthrene.

Inclusion complex formation could also be confirmed by IR spectroscopy. The bands resulting from the encapsulated ‘‘guest’’ molecules are generally shifted or their intensities are altered [45]. The ATR-IR spectra of βCD, PyβCD (**2**), Poly-PyβCD (**3**), Poly-SMP-PyβCD (**4**), and that of after encapsulation with phenanthrene are shown in Figure 4. Hydrogen-bonded –OH groups of the βCD appeared at 3280 cm^−1^ while after its pentynylation (PyβCD, **2**), a new signal due to the appearance of free –OH groups appeared at 3641 cm^−1^. This change indicates that pentynylation of βCD introduced hydrophobic character and βCD molecules were separated from each other leading to formation of “free –OH” groups (Figure 4). Additionally, a very week signal in PyβCD due to –C≡C– appeared at 2120 cm^−1^ but its intensity was very low. After polymerization, the signal at 3641 cm^−1^ (due to free OH) was broaden due to the appearance of new kind of free –OH groups. The IR spectrum (Figure 4) of the Poly-SMP-PyβCD (**4**), and that after creating inclusions with phenanthrene, look similar (except a few changes) and is considered a major characteristic of the βCD host-guest inclusion complex as described by Li et al. [46]. Along with expected signals for Poly-SMP-PyβCD, the characteristic band for phenanthrene appeared between 2800–3200 cm^−1^ in the phenanthren-absorbed Poly-SMP-PyβCD [47]. The signals of βCD in Poly-SMP-PyβCD (**4**) are characterized by intense bands at 3343 cm^−1^ due to –OH stretching vibrations in the primary (C-6–OH) or in the secondary –OH groups linked by the intramolecular H-bonds (C-2–OH of one glucopyranose and C-3–OH of the adjacent glucopyranose unit) [12,45]. After creating the inclusion complex with phenanthrene, this band was shifted to 3311 cm^−1^ due to the involvement of the –OH groups with phenanthrene molecules. In the region of 1400–1200 cm^−1^, the absorption bands at 1425, 1404, 1356, 1319, 1282, 1235, and 1192 cm^−1^ due to the deformation vibrations of the C–H bonds in the primary and secondary OH groups in βCD were also shifted, respectively, to 1422, 1401, 1344, 1317, 1378, 1228, and 1178 cm^−1^. In the region of 1200–1030 cm^−1^, the absorption bands of the valence vibrations of the C–O bonds in the ether and –OH groups of the βCD were shifted from 1096 to 1055 and 1049 to 1031 cm^−1^. The region from 950 to 700 cm^−1^ in βCD spectrum show the absorption bands at 931, 845, 750, and 701 cm^−1^ belonging to the deformation vibrations of the C–H bonds and the pulsation vibrations in glucopyranose cycle in βCD. In βCD-phenanthrene complex, these bands were shifted, respectively, to 926, 843, 734, and 699 cm^−1^.

Thus, all vibrations and bends of the βCD were shifted to lower frequencies after complexation with phenanthrene. This observation is different from the reported data [45,48] where all vibrations and bends of the βCD were shifted to slightly higher frequencies due to the of the “guest” molecules into the βCD cavity. This difference is due to the difference in the chemical structure of the “guest” molecules, i.e., guest molecules cause the in the vibrations and bend frequencies, but the nature of the shift (blue- or redshift) would depend upon the chemical nature of the guest molecules. These observations are in agreement with the reported literature [49].

### 3.6. Thermal Gravimetric Analysis (TGA)

**Figure 5:**
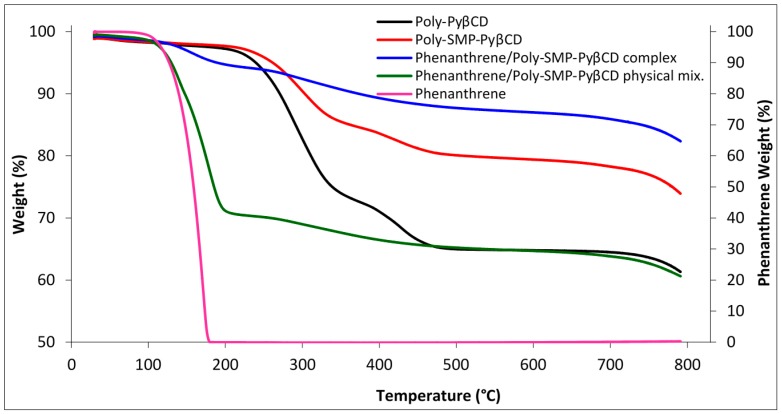
TGA analysis of the Poly-PyβCD, Poly-SMP-PyβCD, phenanthrere/Poly-SMP-PyβCD complex, phenanthrene/Poly-SMP-PyβCD physical mixture and pure phenanthrene. Weight loss of phenanthrene is shown on the right side of the graph while others on the left side of graph.

TGA analyses of Poly-PyβCD and the phenanthrene encapsulated in βCD cavities provides information about the constituents and their quantities present in the composite material. It also allowed us to investigate their degradation kinetics and water loss [18,19,20,21,22]. Results indicate that the enhanced efficiency of the Poly-SMP-PyβCD (**4**) compared to unmodified SMP was due to the inclusion complex formation between the βCD cavity and phenanthrene. For this purpose, TGA was conducted on the samples to identify changes in the weight percent with respect to the increase in temperature. TGA was performed on Poly-PyβCD (**3**), Poly-SMP-PyβCD (**4**), phenanthrene, and after complexation of Poly-SMP-PyβCD (**4**) with phenanthrene for the temperature range of 30 to 800 °C, and the results are plotted in Figure 5. Poly-PyβCD (**3**) exhibits two separate weight losses areas; first, from 40–200 °C due to the loss of water molecules attached with OH by H-bonding in the βCD cavity, losing 1.96% weight, and second, from 200–345 °C due to the decomposition of the βCD macrocycles and 23% of the original weight was lost in this region. Along with two major phases, there was also a third, and a bit slower, phase ranging from 345 to 512 °C, losing 9% of the weight. Poly-SMP-PyβCD (**4**) remained stable until 207 °C and only 1.64% weight was lost due to moisture evaporation. This indicates that thermal stability of Poly-PyβCD (**3**) was slightly improved after hybridization with SMP. Phenanthrene remained almost stable until 101 °C (the melting point of phenanthrene) and only 1.24% weight was lost due to moisture removal. After that, it degraded very rapidly and almost 100% weight was lost from 101 to 183 °C. The Poly-SMP-PyβCD/phenanthrene complex remained stable until 112 °C, and only 1% weight, probably due to the evaporation of moisture present in the sample, was lost. This indicates that encapsulation of the phenanthrene in βCD cavity has improved its thermal stability and is in agreement with the reported data [36,37]. Similar observations have been reported by Narayanan et al. [21] for encapsulating of poly(ε-caprolactone) in α-cyclodextrin.

In comparison, thermal analysis was also done on the phenanthrene/Poly-SMP-PyβCD physical mixture and pure phenanthrene to confirm that the improvement in phenanthrene stability was due to its inclusion complex with βCD. The physical mixture also remained stable until 101 °C, followed by rapid degradation, and 27% of the weight was lost from 101 to 197 °C followed by Poly-PyβCD degradation (197–340 °C). The main degradation in pure βCD was started at 200 °C, in Poly-SMP-PyβCD (**4**) at 207 °C, after making complexation with phenanthrene at 253 °C, while in the physical mixture, it started again at about 200 °C. In the same way, degradation of pure phenanthrene and in the physical mixture was started at 101 °C, while that of the inclusion complexation was started at 112 °C. These results indicate that the formation of the inclusion complex has changed the thermal degradation properties of βCD and phenanthrene, and that the improvement in the phenanthrene removal capacity of the Poly-SMP-PyβCD (**4**) was due to the inclusion complexation of phenanthrene in the βCD cavity.

## 4. Conclusions

A hybrid material of silica micro-particles (SMP) and a polymer of pentynyl βCD (Poly-PyβCD) was prepared and used for the phenanthrene removal from the aqueous solution. Results show that pentynyl groups induce a non-polar character to the βCD polymer and make it insoluble in water. Phenanthrene capturing efficiency of the hybrid material (Poly-SMP-PyβCD) was improved significantly compared to the unmodified SMP. Infrared spectroscopy and thermal gravimetric analysis show that enhanced efficiency was due to the inclusion complexation formed between phenanthrene and βCD cavity. It is probable that the hydrophobic nature of the pentynyl groups introduced on the βCD surface also helps to attract phenanthrene towards the βCD and plays a role in improving the adsorption capacity of the Poly-SMP-PyβCD. These results indicate that Poly-SMP-PyβCD hybrid materials have a potential to be applied as molecular filters in water purification systems and also for waste water treatment.
